# Current state of preconception care in sub-Saharan Africa: A systematic scoping review

**DOI:** 10.4102/phcfm.v14i1.3096

**Published:** 2022-04-26

**Authors:** Winifred C. Ukoha, Ntombifikile G. Mtshali, Lateef Adepeju

**Affiliations:** 1 School of Nursing and Public Health, College of Health Science, University of KwaZulu-Natal, Durban, South Africa

**Keywords:** preconception care, scoping, knowledge, utilisation, provision, sub-Saharan Africa

## Abstract

**Background:**

Preconception care (PCC) utilisation is essential to extend and complete the health continuum. However, these services are not yet incorporated into many low-income countries’ existing maternal health services.

**Aim:**

This study aims to review the current literature on the knowledge, utilisation and provision of PCC.

**Setting:**

This included women and healthcare workers (HCWs) in Sub-Saharan African (SSA) countries.

**Methods:**

Arksey and O’Malley’s scoping review methodology framework is used in this study. The following databases, Google Scholar, Science Direct, PubMed, Scopus and Dissertation via ProQuest, were searched. Articles that met the eligibility criteria were included in this study.

**Results:**

Out of the 451 retrieved articles, 39 were relevant. In most studies, women’s utilisation and HCW’s provision of PCC were considered limited. Their knowledge, however, varies between studies, and there were a few studies conducted among women with chronic conditions. Several factors influenced women and HCWs’ knowledge, utilisation and provision of PCC, including age, level of education, employment, practice area, resources and knowledge. Preconception care interventions most commonly identified, utilised and provided were HIV testing, counselling and family planning, while preconception folic acid supplementation was the least.

**Conclusion:**

The estimates of knowledge and utilisation were suboptimal among women, while provision was the worst affected among HCWs. Gaps exist between the HCW knowledge and practice of PCC. There is a need to promote, prioritise, integrate and optimise the opportunistic provision of PCC in SSA. There is also a need for more studies on PCC provision and utilisation among women with chronic medical conditions.

## Background

Definition of preconception care (PCC) varies from:

[*T*]he provision of biomedical, behavioural and social health interventions to women and couples before conception occurs, aimed at improving maternal and child health outcomes in both the short and long term.^1^

To:

[*A*] set of interventions or programs that aims to identify and enable informed decision-making to modify biomedical, behavioural and (psycho) social risks to parental health and the health of their future child – through counselling, prevention and management, emphasising those factors that must be acted on before conception and in early pregnancy, for a maximal impact and choice.^2^

An older definition by the Centre for Disease Control as ‘a set of interventions which aim to identify and modify biomedical, behavioural, and social risks to maternal health and pregnancy outcome through management and prevention’.^3^ Therefore, it is more than a single visit and less than all well-woman care. It includes care before the first pregnancy or between pregnancies (commonly known as inter-conception care). Similarly, the preconception period has been defined as ‘a minimum of three months before conception’^4^ to ‘a minimum of 1–2 years before the initiation of any unprotected sex that could result in a pregnancy’.^5^ However, a more recent definition by Lancet series proposed the preconception period from:

[*A*] biological perspective as days to weeks before fertilisation. They also looked at it from an individual perspective of weeks to months of a conscious intention to conceive and from a public health perspective of longer periods of months or years to address preconception risk factors like diet and obesity.^6^

Reducing maternal and child mortality and morbidity remains a global priority through the Sustainable Development Goals for 2030.^7^ The need to address preventable mortality causes has emerged. Preconception care is one of the strategies for addressing the preventable causes of maternal and child deaths at the grassroots level and improving birth preparation and the mother’s overall health. However, it has received little attention in low- and middle-income countries (LMICs).^4^ As a strategy for preventing maternal and child mortality, PCC targets the aspects of PCC counselling, risk assessment and interventions, which ensure that women enter pregnancy in a good state of health. Preventive care has been confirmed to reduce healthcare costs in other regions like the United States.^8^ The integration of PCC has proved feasible for both the high- and low-income countries.^1^ This care should be a priority for women of childbearing age with chronic conditions. However, most LMICs pay less attention because of the competing disease burden.^1^ Preconception care intervention was proven to have long- and short-term effects on mother and child health. Studies revealed that micronutrient supplementations resumed at pregnancy can correct such deficiencies in the mother but do not affect the child’s health outcomes.^6^ Therefore, these supplementations should start before conception for maternal and child benefits.

Women of childbearing age in LMICs, where PCC is most required because of high maternal and child mortality rates, do not receive it, owing to inaccessibility or unavailability.^4^ Recommendations made following a critical assessment of the state of global maternal, neonatal and child health state that LMICs would be the greatest beneficiaries of PCC services, and thus, encourage them to find a cost-effective way of improving access to PCC interventions.^1^ Preconception care interventions are those health interventions delivered before conception to address risk factors, health and behavioural problems, such as nutritional, contraception, control of chronic conditions, mental health, micronutrient supplementation, tobacco use, genetic, vaccination, sexually transmitted infections screening and treatment.^9^ Maternal and child health experts also emphasised the importance of developing strategies to increase access to the most basic PCC interventions, such as nutrition, contraception, prevention and treatment of chronic maternal health conditions, immunisation, adolescent reproductive planning and reducing the harmful effects of smoke exposure in LMICs.^4^ Preconception care interventions include promoting reproductive health, nutritional status and supplementation, preventing and treating infections, screening and managing chronic diseases, substance abuse and lifestyle changes to improve pregnancy outcome.^10^ The WHO suggested that countries conduct feasibility studies to determine the best cost-effective way of integrating PCC into existing maternal and child health services.^1^ However, PCC is not yet provided to all women and couples of childbearing age in several countries worldwide. Healthcare providers hardly discuss its importance and availability with women.^11^

Many maternal and child health priorities in Africa and several other low-income countries are far more pressing than preventive care such as PCC. Thus, several maternal and child health policies emphasise quality and access to prenatal, labour and postnatal care, and PCC guidelines are non-existent in many countries.^12^ Nonetheless, the development and implementation of PCC policies and guidelines are other factors that will ensure that healthcare providers have the information they need to provide PCC.^13^ Some countries in sub-Saharan Africa (SSA) have certain PCC recommendations included in their national guidelines,^14^ while others have no recommendations.^15^

Despite the identified benefits of intervening before pregnancy, the provision of PCC by healthcare providers and its utilisation by couples planning a pregnancy is reportedly low.^12,16^ Preconception care provision and utilisation are challenging to implement and necessitate the efforts of both healthcare providers and clients. Healthcare providers have a significant impact on PCC utilisation. Nonetheless, PCC provision has been low because it is usually provided opportunistically rather than as a routine with a structured process.^12^ Understanding the PCC knowledge of healthcare workers (HCWs) and people of childbearing age is critical. This is because lack of knowledge has been cited as the primary reason for the non-implementation of most evidence-based healthcare practices, as healthcare providers typically do not practise what they do not know.^17^

Notwithstanding that PCC is a broad topic, we are particularly interested in the dimensions of knowledge, utilisation and PCC provision in SSA. There are three PCC reviews in SSA. One targets safer conception strategies for HIV-positive couples. It reveals that safer conception is widely unavailable to HIV-affected couples because of low providers’ knowledge.^18,19^ Two more recent ones were country specific because they were conducted exclusively for PCC knowledge and utilisation among women in Ethiopian studies. They revealed low PCC knowledge and utilisation levels among women. Family planning utilisation, high educational level and antenatal follow-up were significantly associated with PCC knowledge. In the same vein, age and good knowledge of PCC showed significant association with utilisation.^20,21^ The current review aimed to be more inclusive, with no restrictions on setting and PCC provision. As a result, the goal of this review was to systematically map out evidence on the current state of PCC knowledge, utilisation and provision among women and HCWs in SSA in the published literature. Considering the limited number of reviews about general PCC that targets the entire SSA region, this scoping review was concentrated in the region. This review discussed the knowledge, utilisation and provision of the PCC interventions according to WHO, such as nutrition, control of pre-existing conditions, genetic screening and counselling, managing sexually transmitted infections and family planning. The findings from this review will be shared with other researchers and stakeholders in the region. Thus, the study investigates women’s and HCWs’ knowledge, utilisation and provision of PCC in SSA countries.

### Aim of the study

This study investigates the current documented literature on PCC knowledge, utilisation and provision among women and HCWs in SSAn countries. It also aims to identify factors influencing PCC knowledge, utilisation and provision.

### Research question

What is the documented evidence on knowledge, utilisation and provision of PCC among women and HCWs in sub-Saharan Africa? Which factors influence the knowledge, utilisation and provision of PCC?

## Methods

The approach of conducting a scoping review is critical for identifying the current knowledge gap in the field of interest.^22^ This review followed the Arksey and O’Malley and Joanna Briggs Institute scoping review frameworks.^22,23^ This study used five of the six scoping review steps from Arksey and O’Malley’s methodological framework. These steps include identifying the research question, identifying relevant studies, selecting a study, charting the data, collating, summarising and reporting the results. The sixth step was omitted because it is optional. This review followed the Preferred Reporting Items for Systematic Reviews and Meta-Analyses Extension for Scoping Reviews (PRISMA-ScR) checklist and reporting guideline (Online Appendix 1).^24^ This review of studies is part of a larger PhD project. A scoping review protocol was developed; however, it was not registered or published in any journal prior to the start of this review, as per the International Prospective Register of Systematic Reviews (PROSPERO) guidelines. On request, the protocol will be made available.

### Identify a research question

We identify the central research question that relates to the study’s purpose. What has been documented in the existing literature about women’s and HCWs’ knowledge, utilisation and provision of PCC in SSA countries? This was performed to assist us in identifying the gap in our knowledge of this phenomenon. The Joanna Briggs Institute Reviewer’s Manual, 2015^23^ for the Population, Concept and Context, as shown in Table 1, was used to determine the study’s eligibility criteria.

**TABLE 1:** Framework for the determination of eligibility of the review question.

Criteria	Determinants
Population	Healthcare workers (professional nurses, doctors, pharmacists, any gender and age) and people of childbearing age, including women, men and adolescents (any age from 10 to 49 years). These are the providers who should benefit from PCC.
Concept	Preconception care OR preconception health
Context	Sub-Saharan African country grouping according to African Union, including Sudan.

*Source:* Adapted form Peters M, Godfrey C, McInerney P, Soares CB, Khalil H, Parker D. Methodology for JBI scoping reviews. The Joanna Briggs Institute reviewers manual 2015. Adelaide: The Joanna Briggs Institute, 2015; p. 3–24.

PCC, preconception care.

### Identify relevant studies

**FIGURE 1:**
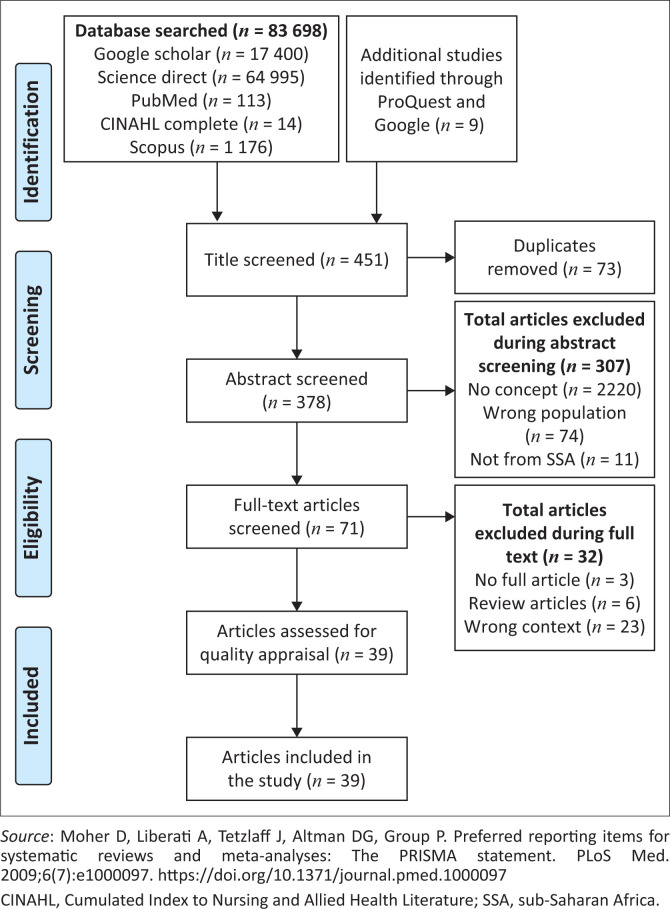
Adapted preferred reporting items for systematic reviews and metaanalyses flow chart, demonstrating search and selection of studies.

This study included empirical studies as well as grey literature such as academic dissertation documents, including government and non-governmental organisation documents. This stage consists of screening study titles relevant to the topic, abstracts and full articles. The following electronic databases were used to look for peer-reviewed articles that answered the review question; Google Scholar, Science Direct, PubMed Central, CINAHL Complete and Scopus. Articles published in English between January 2011 and May 2020 were included in the study to provide more recent secondary data. The title screening was carried out solely by the principal investigator (W.C.U.), who exported the results into a newly created Endnote library. After that, duplicates were removed before involving a co-screener (P.A.) in the study. During title screening for the study, the search strategy Medical Subject Headings (MeSH) terms such as PCC/pre-pregnancy care/inter-conception care; knowledge/awareness/; utilisation/provision/practice, and uptake was used during the search. Flowchart of preferred reporting items for systematic reviews and meta-analysis as shown in Figure 1,^25^ was used to report the number of records retrieved and those included and excluded.

### Study selection

A pilot study was conducted using keywords from the study population, concept and context to determine the appropriateness of the database chosen. In addition, the university librarian’s assistance was sought for difficult-to-access articles, and the authors were contacted for articles that were not available through electronic search. An End Note library, data management software, was created for this review. All study designs were included in this review. The inclusion of all elements of the research questions ensured that the study’s eligibility criteria were met. Two reviewers independently screened the abstracts and full text, and a third reviewer resolved the discrepancies.

### Inclusion and exclusion criteria

Articles were included in this review if they met the following criteria: (1) studies on PCC and its synonyms in the SSA context; (2) if the paper was published in English and was available in full-text; (3) studies on PCC, regardless of design, including quantitative, qualitative and mixed studies, and (4) the grey literature on PCC from governmental and non-governmental organisations, as well as academic dissertations.

### Data extraction

The goal of the data extraction process in a scoping review is to create a descriptive summary of the findings. Online Appendix 1 shows the extracted data and the process used to extract it. Relevant data from the included studies were charted, and all interventions were compared. The research team created a data charting form and determined what was charted in this study. The extraction of data and charting of data from the eligible studies was carried out independently by the principal investigator. Following that, the research team met to determine whether the data extraction approach and research purpose were consistent, as recommended by^23^ Braun and Clarke 2006 guiding steps.^26^

As a result, the descriptive-analytical method was used in this review, which entails applying an analytical framework to all research reports and collecting standard information on each study.^22^ Data were sought about knowledge, utilisation, and provision of PCC and factors associated with knowledge, utilisation and provision of PCC.

### Critical appraisal

As previously demonstrated, the lack of methodological quality assessment makes interpreting scoping review results difficult.^27^ As a result, the Mixed Methods Appraisal Tool (MMAT) version 2018 was used for quality assessment to assess the methodological qualities of the included studies.^28^ Depending on the study design, different sections of the MMAT were used. The methodology, design, aim, data collection, data analysis, results and conclusions of all included articles were used to assess their quality and appropriateness.

Two independent reviewers critically appraised all papers included in this study. The appraisal checklist scores were used to rate the quality of the included papers on a three-point scale as high, medium or low. As a result, no paper was rejected based on this rating system. The scores were used as a variable in the analysis stage. The number of ‘yes’ responses for each paper’s methodological criteria was counted and computed to generate a percentage score. Scores of less than 50% were considered low; scores from 51% to 75% were considered average, while 76% or higher scores were considered high. The methodological quality appraisal of the majority of included studies was high, ranging from 80% to 100%, with only two studies scoring average, at 70% and 65%, as shown in Online Appendix 1. As a result, 37 of the included studies can influence PCC policy and service delivery.

### Collating, summarising and reporting the result

The location, methodologies, categories measured and findings of the studies were all described. Evidence was synthesised using the following categories: (1) knowledge and awareness of PCC services, (2) factors associated with the knowledge and awareness of PCC services, (3) utilisation and provision of PCC services, and (4) factors influencing utilisation and provision of PCC services. The scope of recent research on PCC knowledge, utilisation and provision in SSA was mapped and summarised.

### Ethical considerations

Ethical clearance for the study was obtained from the University of KwaZulu-Natal Human and Social Sciences Research Ethics Committee – HSSREC/00001069/2020.

## Results

The overview and categorical analysis of the included studies are presented in Online Appendix 1. From the 451 articles retrieved from six electronic databases and nine from other sources, only 39 (32 from primary sources and seven grey literature) met the study’s inclusion criteria and were included for the data extraction. The Cohen inter-rater reliability analysis using kappa’s statistic was performed to determine the degree of agreement between the screeners at the full-text screening stage. The agreement level among the two reviewers was 85.4%, which showed an adequate agreement level (Kappa statistic = 0.62, *p* < 0.000). McNemar’s chi-square statistic indicates no statistically significant difference in the proportions of yes and no responses among the reviewers.

### Characteristics of included studies

Online Appendix 1 shows the characteristics of the included studies, revealing that 16 of the 39 studies in this review were from Ethiopia,^15,29,30,31,32,33,34,35,36,37,38,39,40,41,42,43^ 11 from Nigeria,^44,45,46,47,48,49,50,51,52,53,54^ four from Kenya,^55,56,57,58^ two from Sudan,^59,60^ two from Zimbabwe,^61,62^ one from Malawi,^63^ one from South Africa,^64^ one from Swaziland^65^ and one from Zambia.^66^

Thirty-five (35) studies^15,29,30,31,32,33,34,36,37,38,39,40,41,43,44,45,46,47,48,49,50,51,52,53,54,56,57,58,59,60,61,63,64,65,66^ used a quantitative descriptive design; three used a mixed-method approach^34,42,55^ and one used a qualitative study design.^62^ Only knowledge of PCC was assessed in 18 of the reviewed articles.^15,31,32,38,40,41,42,44,49,51,53,54,56,57,60,61,62,64^ Fourteen studies assessed both knowledge and utilisation of PCC,^29,34,35,36,43,45,46,47,48,50,59,63,65,66^ four studies assessed only utilisation,^30,37,55,58^ two studies assessed both knowledge and provision^33,52^ and one study assessed the provision of PCC.^39^ None of the studies in this review engaged with men’s knowledge or utilisation of PCC. Twenty-nine articles examined PCC knowledge among women, seven examined PCC knowledge among healthcare workers and 17 examined PCC utilisation among women. Only one article assessed PCC knowledge in both women and healthcare workers. Three articles reported PCC provision or practice among HCWs. Only one article assessed PCC knowledge in both women and healthcare workers. Women with chronic medical conditions such as rheumatoid heart disease^59^ and diabetes^42,66^ had their PCC knowledge and utilisation assessed.

### Operational definition of terms

Preconception care for this review is any interventions, be it screening, advice, treatment and lifestyle modification provided to people of childbearing age before conception. Components of PCC in the reviewed articles are nutrition, micro-nutrient supplementation, human immunodeficiency virus (HIV) testing and management, smoking and alcohol cessation, immunisation, screening for environmental toxins, chronic condition management, genetic condition, mental health and contraception.

Awareness of PCC was measured mainly by awareness of PCC service availability.

Preconception care knowledge was measured mainly by identifying its components and sometimes using the definition of PCC, its concepts and timing. Identification of more than 50% of PCC components was considered good or high knowledge and less than 50% as poor or low knowledge.

Utilisation of PCC was mainly assessed by the uptake of any interventions, either advice, screening, treatment or lifestyle modification, at least once before conception.

### Knowledge/awareness of preconception care

Most of the reviewed articles (*n* = 20) used the WHO definition of PCC, and two used the CDC definition. The assessment of PCC knowledge across the papers was mainly based on PCC components’ knowledge. However, awareness of PCC generally assesses whether women know about various PCC services or the concept. The elements included in the PCC knowledge were awareness of PCC concepts and services, source of information, sometimes the definition and timing of PCC and knowledge of PCC components. The PCC interventions included in the review are (1) folic acid and micronutrient supplementation, (2) control of pre-existing conditions, (3) genetic counselling, (4) HIV testing and (5) family planning. These interventions are the PCC components mainly discussed in the reviewed articles.

The PCC knowledge estimates range from 11% to 91.2% among women of childbearing age and 23% – 90% among HCWs. Twenty-one of the 29 studies that assessed PCC knowledge among women reported it to be low. In 14 of the studies reviewed, healthcare workers were the primary source of information for women who were aware of PCC services, followed by media in three^38,45,61^ and lectures among students.^53^ The knowledge of PCC concepts, definitions and timing among women was so low that some mistook it for antenatal care as they were unaware of PCC timing.^58,62^ Out of the seven reviewed studies that assessed PCC knowledge among HCWs, five reported a good level of knowledge.^32,43,44,52,64^ In contrast, two reported a low level of knowledge.^15,33^ Some HCWs could correctly define PCC and know when to provide PCC services to women and couples.^44,52^ The HCW’s knowledge of PCC services components was reported to be as high as 90% in some cases^15,44,64^ and only slightly higher in others.^32,52^ In some studies, more than 83% of HCWs were aware of the benefits of providing PCC services to the general population.^52,64^ However, there was some confusion about who should provide these services.^52^ They also claimed that they were not taught about PCC services during their training period and did not receive any in-service PCC education.^33,52^ Some PCC components, such as pre-marital genetic counselling^53^ and preconception folic acid supplementation,^38,49^ were assessed as a single component among women.

The knowledge of preconception folic acid supplementation was among the least commonly identified PCC components among women of childbearing age.^47^ Most women believe that folic acid and other micronutrient supplementation should only be given after pregnancy is confirmed. They were not able to identify the required dose of folic acid during the preconception period and pregnancy. The knowledge of genetic screening and counselling before marriage and in the preconception period was not recognised in most of the reviewed articles except when assessed as a single component. HIV testing, counselling and management^36,47,61,62^ were the most commonly identified PCC service components among women. The knowledge of this component was high in all the reviewed articles. Another component of PCC that most women identified is family planning and contraception.

### Factors associated with the knowledge of preconception care among women and healthcare workers

Women with a higher level of education know more about PCC than women with a lower level of education.^31,35,36,38,40,42,47,49,50,51,59,66^ Knowledge of PCC increases with age and parity, with older women and those with more parity being more knowledgeable than others.^31,51,56^ Similarly, diabetic women who have diabetic follow-up and those diagnosed with diabetes for a more extended period^42,66^ have a high level of knowledge of PCC. Women who work in the formal sector have higher PCC knowledge than others.^35,42,49,54,63^ Those studying in the health department and those living in cities^40,47,53^ are also more knowledgeable about PCC. Women’s PCC knowledge is associated with scheduling ANC early and having antenatal and postnatal contact.^35,40,49^ Increased knowledge of PCC is associated with a history of family planning use or use of modern contraceptives and hospital delivery.^31,35,38,47^ Preconception care knowledge is associated with exposure to PCC counselling alone or with a partner and having a husband’s support or healthcare workers as a source of PCC information.^29,59^ Women’s PCC knowledge is also associated with a history of adverse pregnancy outcomes and chronic conditions.^29,38^ Knowledge of PCC is also associated with using a radio or phone and having a higher household income.^38,40^ Women who are Christians or visit specific healthcare facilities while pregnant have a higher level of knowledge than others.^44,63^

These are some of the factors associated with PCC knowledge among healthcare workers in the identified studies. Healthcare workers who work in hospitals, rural areas or clinics typically have more knowledge than those in other settings.^15,32,64^ Working in a practice where PCC guidelines are used, having read a PCC guideline and using a smartphone to access clinical resources are all associated with PCC knowledge.^15,32,33^ Knowledge of PCC is also associated with training in PCC education and counselling or HIV testing and management.^32^ Healthcare workers who have participated in PCC public awareness and have provided PCC have greater PCC knowledge than others.^15,32,33^ Preconception care knowledge was associated with fewer years of experience and age.^52,64^ Healthcare workers who specialise or work in obstetrics and gynaecology practice and those who earn a higher salary are more knowledgeable about PCC than the rest.^15,33,43^

### Utilisation and provision of preconception care services

The PCC utilisation estimates range from 8.1% to 86.8% among women of childbearing age, while PCC provision by HCWs ranges from 15.3% to 47.7%.

Out of the 17 articles that reported PCC utilisation among women, 15 reported low utilisation.

Preconception micronutrient^29,30,52^ and folic acid supplementation were among the least commonly utilised PCC components among women.^35,37,55^ In contrast, folic acid supplementation was the most commonly reported intervention provided by HCWs.

Preconception care interventions use was low amongst women with chronic conditions such as diabetes and rheumatic heart disease.^59,66^ Most of them were not counselled about their condition, and many preconceptions’ screening was not conducted.

Human immunodeficiency virus testing, counselling and management were among the most common provided PCC interventions among HCWs,^34,47,55^ and the most utilised component among women. Family planning and contraceptive services^35^ were among the most commonly used PCC components. However, some women with some chronic medical conditions such as rheumatic heart disease and diabetes did not utilise this service as required. Some women stated that they did not use PCC services because they were unaware of their availability and importance.^50,58,65^ The service was inaccessible,^50^ and the cost was prohibitive.^50^ The majority of HCWs believe that opportunistic delivery is the best mode of PCC service delivery.^52^ Some HCWs did not consider PCC a priority, although they are responsible for its provision.^43^ Most PCC components were either not practised or were practised infrequently.^33,39,43,52^ Some studies^33,43,52^ identified barriers to the provision of PCC services, such as unplanned pregnancies and a lack of awareness among women.

### Factors that are associated with the utilisation and provision of preconception care services among women and healthcare workers

Some of the factors associated with the use of PCC services by women were also identified. Five studies^29,30,34,35,45^ found that having good knowledge of PCC services and awareness of the PCC unit’s availability are related to PCC utilisation among women. Women who had a bad birth experience and those with chronic conditions tend to use PCC more than the rest.^29,30,55^ Women who are married and receive support from their partners are more likely to use PCC than others.^29,30,34,46,55^ The greater a woman’s age and parity, the more likely she will use PCC.^34,37,55^ Women with a higher level of education or professional background and higher family incomes use PCC more than their counterparts.^35,37,47,55,63^ Women planning a pregnancy and those with a history of postnatal care use PCC more than others.^35,37^ The cost of PCC services influences utilisation.^53,55^

Regular screening of the patient’s reproductive life plan^33^ is associated with PCC practice among HCWs. Being a nurse and having little PCC knowledge reduce the chances of providing PCC to women and couples.^39^ Believing that PCC is only for a specific group of professionals rather than all healthcare professionals decreased the likelihood of HCWs providing PCC.^39^

## Discussion

This review summarises evidence of PCC knowledge, utilisation and provision in SSA, as well as the factors influencing these outcomes. Notwithstanding that the SSA region bears most of the global burden of maternal and child mortality, knowledge, utilisation and provision of PCC among women and HCWs are not well studied. Many countries in the region were not represented. The reviewed studies were limited to nine countries (Ethiopia, Nigeria, Kenya, Sudan, Zimbabwe, Zambia, South Africa, Malawi and Swaziland), with only Ethiopia and Nigeria accounting for 69.2% (*n* = 27). There is also methodological variation in the reviewed studies, with 89.7% (*n* = 35) employing a quantitative approach and only 2.5% (*n* = 1) employing a qualitative approach.

### Preconception care knowledge among women and healthcare workers

In 72.4% of the studies reviewed, women had little knowledge of PCC. In some cases, these women have never heard of the PCC concept, and some even believe that PCC is synonymous with ANC.^49,57^ Women did not know the correct timing of PCC because of their lack of knowledge of the concept.^46,66^ In general, 71.4% of the reviewed studies found that HCWs had good PCC knowledge. The majority of them understood why and when PCC should be provided.^44,52,64^ In 42.8% of the studies reviewed, HCWs knew PCC components.^15,43,44^ However, some HCWs stated that their training did not adequately prepare them to provide PCC with all necessary knowledge. They are also not regularly updated about PCC through in-service education.^49^ However, the majority of them are eager to provide PCC services.^52^ As a result, all necessary training should be provided to HCWs to improve their knowledge, as there is a significant gap between their knowledge and practice of PCC.

Given the high rates of anaemia in SSA, where close to 90% of women become anaemic during pregnancy,^67^ women, especially those in vulnerable areas, should be made aware of the need for preconception supplements. However, women in the reviewed studies had little knowledge of preconception supplements. The knowledge of preconception folic acid use among women was lacking.^37,56,57^

When women are prescribed folic acid during pregnancy, HCWs rarely mention the indication to them, as a result. Some people are unfamiliar with the name and can only identify it based on its description.^58,62^

The knowledge about pre-marital genetic counselling and genetic counselling, in general, was limited to women except in one study. Despite this, knowledge of HIV testing and management were the most identified PCC components among women,^36,47,53,61,62^ which may be because of the continent’s high rate of HIV. Safer conception is the PCC delivery strategy widely used by all countries in SSA. Articles addressing safe conception in the continent were not included in this review paper because safer conception is a broad topic in SSA and has previously been discussed in a review.^19^

In the reviewed studies,^35,56^ women’s knowledge of family planning as a PCC component was low. The concept of a reproductive life plan, which also forms part of the PCC strategy of ensuring pregnancy planning,^3^ was absent.

### Preconception care utilisation and provision

In 88.2% of the studies reviewed, PCC utilisation was low. In some studies, preconception folic acid use among women was as low as 0.0%.^37^ Many women are only put on folic acid supplements once pregnancy is confirmed. This is why food and grain fortification are highly recommended in SSA, as most pregnancies are unplanned,^68^ so it is hard to know when women can be placed on these supplements. Women’s main reason for their non-utilisation of PCC services was the lack of knowledge and awareness.^58,66^

According to WHO, PCC should be provided to all couples of childbearing age, considering pregnancy, particularly those with pre-existing chronic conditions.^1^

Nonetheless, women with diabetes in SSA who had previously experienced obstetric disorders because of uncontrolled diabetes did not receive some of the necessary assessments, such as frequent blood sugar monitoring.^60^ In another study, most women with pre-existing diabetes did not seek PCC because they were unaware that they were required to do so. Others did not believe that PCC was necessary. The remainder were afraid that the HCWs would discourage them from becoming pregnant.^66^ More than half of women with rheumatic heart disease did not use contraception and were not counselled about becoming pregnant. Furthermore, even after explaining the PCC concept and its benefits, many women stated that they do not intend to seek PCC during their subsequent pregnancy.^59^

Genetic screening and counselling are essential parts of PCC strategies, especially in some areas with serious genetic conditions to improve the children’s overall health in the long run.^69^ Genetic counselling was hardly identified or utilised as a PCC component in the current review. HIV testing, counselling and management, and family planning were the most utilised PCC interventions in SSA from the reviewed articles.

Preconception care provision was low among HCWs because of role uncertainty and a lack of resources or guidelines to remind HCWs. Some PCC components, such as vaccination, folic acid supplementation and weight management, were non-existent or as low as 0% in some studies.^33,39^ The HCWs rarely enquire about the clients’ reproductive life plans. Women’s use of PCC can be increased through public awareness and education campaigns. To increase PCC service provision and utilisation in LMICs, strategies should be integrated into existing maternal and child health programmes using a system-based approach. Increasing utilisation and uptake of PCC interventions, particularly among adolescents, improving PCC delivery, collaborating with media and information technology, and utilising community health workers.^1^ As PCC provision goes beyond maternal and child services, its integration into every service that women and men of reproductive age attend such as HIV, medical, post-abortion and postnatal clinics is important. In addition, purposively maximising facility contact will be critical in ensuring PCC provision.

### Factors associated with preconception care knowledge and utilisation among women

The following factors were identified in most reviewed studies regarding PCC knowledge, utilisation and provision among women and HCWs: age-related factors, education-related factors, knowledge-related factors, socio-economic-related factors, experience-related factors, previous-activity or history-related factors and resources-related factors.

According to the studies reviewed, among the age-related factors, PCC knowledge and utilisation among women increase with age and parity.^31,51,56^ This could be because of an increase in the number of contacts with HCWs that comes with increasing age and parity. Higher education or professional status is associated with increased PCC knowledge and utilisation among women.^31,35,36,38,40,42,47,49,50,51,59,66^ This may be because this group can read and access a wide range of information online. In other words, when delivering education campaigns, less educated women must be targeted. Women are more likely to use PCCs when more knowledgeable about them. Employment status and income are two other factors influencing women’s knowledge and utilisation. Women with formal employment and higher income have higher level of knowledge and utilisation of PCC services than others^35,42,49,54,63^ as these women tend to be more health-conscious. Women who have had an unfavourable pregnancy outcome and those with chronic conditions have a higher level of PCC knowledge and utilisation than the rest.^29,38^ This is possibly because of their quest for answers and to avoid the risk of recurrence.

Furthermore, a history of antenatal care attendance, prior postnatal contact, family planning use and utilisation of modern contraceptives, exposure to PCC counselling, chronic medical condition and previous hospital delivery were linked to higher PCC knowledge.^31,35,38,47^ These may be associated with women with better health-seeking behaviour generally. In other words, most factors that expose women to more HCW contact increase their chances of learning about PCC.

### Factors associated with preconception care knowledge and provision among healthcare workers

It was also discovered that among HCWs, age and years of experience related to the number of years spent in the healthcare environment, are also related to PCC knowledge. However, on the contrary, younger HCWs had more PCC knowledge than older ones.^52,64^ Younger people were trained in a more digitalised world, and their access to knowledge may differ from that of older HCWs. Factors such as employment area are linked to PCC knowledge among HCWs. Those working in hospitals, clinics and rural areas have more knowledge.^15,32,64^ Some of these HCWs encounter more complications of not utilising PCC, while others practising as independent practitioners appreciate it more without shifting the responsibility to the next person. Working in or having a specialty in obstetrics and gynaecology is also associated with PCC knowledge, which is in keeping with targeted training received in the discipline. Previous PCC training and counselling, HIV testing and management training, and PCC public awareness participation are linked to PCC knowledge and provision among HCWs.

Similarly, HCWs who regularly screen their patients’ reproductive life plans are more likely to provide PCC. Preconception care resources should be available and accessible to increase PCC knowledge of HCWs. The presence and access to guidelines among HCWs increased their PCC knowledge.^15,32,33^ Therefore, this indicates the need to provide and scale up access to PCC guidelines for HCWs.

With the factors mentioned above associated with HCW knowledge, utilisation and provision of PCC and women of childbearing age, all PCC stakeholders must know what and whom to target to improve this dimension of PCC. According to the World Health Organisation, PCC should target socially and economically marginalised people in society.^1^ This review identified some social and economic factors influencing PCC knowledge and utilisation.

This review included the knowledge, utilisation and provision of PCC interventions such as preconception folic acid and micronutrient supplementation, control of pre-existing conditions (diabetes and cardiac conditions), genetic counselling, testing and managing sexually transmitted infections and family planning.

### Implication and recommendations

The only aspect of PCC that has been reviewed in SSA is strategies for safer conception among people living with HIV. This review identified and summarised knowledge, use and provision of PCC services for other chronically ill women and the general population. It reveals knowledge, utilisation and provision gap and factors that positively affect them among women and HCWs. It focuses on the major stakeholders’ level of knowledge and utilisation of the service.

According to this review, further qualitative studies on the knowledge, utilisation and provision of PCC among women and HCWs in SSA are required. Furthermore, based on the results of the included studies, there is a need for interventions to improve PCC knowledge and utilisation in the SSA region. Massive public awareness campaigns and education about the benefits of PCC are critical, as research has shown that knowledge of PCC is associated with both utilisation and provision. This educational campaign should involve all stakeholders and use a different communication level. This information can be disseminated to the public through media and technology. This will ensure the knowledge and utilisation of other PCC interventions such as folic acid and micronutrient supplementation, genetic counselling, vaccination, alcohol and smoking cessation, weight control, mental health and environmental screening. The provision and utilisation of PCC among women at risk of adverse pregnancy outcomes, particularly those with chronic conditions, require additional research. Furthermore, countries should find ways to incorporate PCC services into existing routine care to avoid the additional burden of a dedicated unit on both human and financial resources.

As HCWs’ knowledge and practice of PCC are not yet optimal, there is a need for HCW training and education. This can take the form of in-service education, workshops and seminars to emphasise the importance of providing this service to those working in clinical settings. There is a need to incorporate PCC into the curriculum of HCWs who are still in training institutions.

It is critical that institutions develop PCC guidelines, protocols and policies and readily avail them for HCWs. This will guide and remind HCWs about PCC provision and serve as a constant reminder that women’s care and management should begin prior to conception. A single reproductive life plan question should be included routinely in the clinical assessment of women and men of childbearing age. Similarly, PCC is for both men and women and should not be considered the responsibility of women solely. Food fortification should be intensified to ensure that women get enough micronutrients.

The current study’s limitations are that studies defined and rated PCC knowledge differently, thus hindering the findings’ general ability. Studies on PCC knowledge and utilisation were conducted among women only. Studies on men’s PCC knowledge and utilisation would have contributed additional knowledge. The included studies were heterogeneous in collected data, and therefore, consistent conclusions among studies were difficult. Another limitation is that the review was conducted on studies on PCC in SSA between 2011 and 2020.

## Conclusion

This review outlined the knowledge, utilisation and provision of PCC in SSA, as well as the factors associated with the knowledge, utilisation and provision of PCC. Estimates of knowledge, utilisation and provision among women and HCWs were all suboptimal, with utilisation and provision being the worst affected because the most common PCC components, such as folic acid supplementation and family planning, were underutilised. There is, however, a gap between HCW knowledge and practice of PCC, as most of them are highly knowledgeable about PCC but practise it infrequently. The findings of this review have revealed the current knowledge, practice and utilisation of PCC in SSA and identified the factors associated with knowledge, provision and utilisation to target the appropriate people needed to improve preconception services. There is a need to promote, integrate and prioritise PCC in SSA. As opportunistic provision seems to be the most feasible strategy, this should be optimised to ensure effective provision. The identified enabling factors should be promoted, while hindrance to knowledge, utilisation and provision should be addressed for effectiveness. There is a need for more PCC studies in the unrepresented settings using a variety of methodologies and more studies among women with chronic high-risk conditions.
