# Erratum: Current state of preconception care in sub-Saharan Africa: A systematic scoping review

**DOI:** 10.4102/phcfm.v15i1.3708

**Published:** 2023-11-28

**Authors:** Winifred C. Ukoha, Ntombifikile G. Mtshali, Lateef Adepeju

**Affiliations:** 1 School of Nursing and Public Health, College of Health Science, University of KwaZulu-Natal, Durban, South Africa

In the published article, Ukoha WC, Mtshali NG, Adepeju L. Current state of preconception care in sub-Saharan Africa: A systematic scoping review. Afr J Prim Health Care Fam Med. 2022;14(1): a3096. https://doi.org/10.4102/phcfm.v14i1.3096, an omission error occurred in the online-only Online Appendix 1 (supplementary data). Table 1-A1, entitled ‘Summary of included studies examining preconception care knowledge, utilisation, and provision in sub-Saharan Africa’, was initially omitted from the online-only Online Appendix 1 on the first publication. The updated and correct online-only Online Appendix 1 replaced the incorrect version with its republication.

The publisher apologises for this error. The correction does not change the study’s findings of significance, the overall interpretation of the study’s results, or the scientific conclusions of the article in any way.

